# Nitric Oxide Improves the Tolerance of Pleurotus ostreatus to Heat Stress by Inhibiting Mitochondrial Aconitase

**DOI:** 10.1128/AEM.02303-19

**Published:** 2020-02-18

**Authors:** Ludan Hou, Mengran Zhao, Chenyang Huang, Xiangli Wu, Jinxia Zhang

**Affiliations:** a Institute of Agricultural Resources and Regional Planning, Chinese Academy of Agricultural Sciences, Beijing, China; b Key Laboratory of Microbial Resources, Ministry of Agriculture and Rural Affairs, Beijing, China; The Pennsylvania State University

**Keywords:** *P. ostreatus*, aconitase, nitric oxide, heat stress, oxidative damage

## Abstract

Pleurotus ostreatus is widely cultivated in China. However, its cultivation is strongly affected by seasonal temperature changes, especially the high temperatures of summer. Nitric oxide (NO) was previously reported to alleviate oxidative damage to mycelia by regulating trehalose. In this study, we found that NO alleviated oxidative damage to *P. ostreatus* mycelia by inhibiting the protein and gene expression of aconitase (ACO), and additional studies found that the overexpression and interference of *aco* could affect the content of citric acid (CA). Furthermore, the addition of exogenous CA can induce alternative oxidase (*aox*) gene expression under heat stress, reduce the content of H_2_O_2_ in mycelium, and consequently protect the mycelia under heat stress. An additional analysis focused on the function of the *aox* gene in the heat stress response of mycelia. The results show that the colony diameter of the *aox* overexpression (OE-*aox*) strains was significantly larger than that of the wild-type (WT) strain under heat stress (32°C). In addition, the mycelia of OE-*aox* strains showed significantly enhanced tolerance to H_2_O_2_. In conclusion, this study demonstrates that NO can affect CA accumulation by regulating *aco* gene and ACO protein expression and that CA can induce *aox* gene expression and thereby be a response to heat stress.

**IMPORTANCE** Heat stress is one of the abiotic stresses that affect the growth and development of edible fungi. Our previous study found that exogenous NO had a protective effect on mycelia under heat stress. However, its regulatory mechanism had not been elucidated. In this study, we found that NO altered the respiratory pathway of mycelia under heat stress by regulating *aco*. The results have enhanced our understanding of NO signaling pathways in P. ostreatus.

## Abstract (precis)

Heat stress is one of the abiotic stresses that affect the growth and development of edible fungi. Our previous study found that exogenous NO had a protective effect on mycelia under heat stress. However, its regulatory mechanism had not been elucidated. In this study, we found that NO altered the respiratory pathway of mycelia under heat stress by regulating *aco*. The results have enhanced our understanding of NO signaling pathways in P. ostreatus.

## INTRODUCTION

Heat stress is an abiotic stress that causes irreversible damage to the growth and development of organisms when the temperature exceeds the critical threshold for a long enough time (1). Heat stress can cause oxidative reactions, membrane lipid peroxidation, and protein degradation in plants (2). High temperature is one of the abiotic stresses that influence the cultivation of Pleurotus ostreatus. Previous studies have shown that heat stress can slow mycelial growth, leading to the excessive production of reactive oxygen species (ROS) in the mycelium and oxidative damage (3). Previous studies have focused on the effects of the damage caused by heat stress, including the effects on fungal growth or the induction of cell death (4). In recent years, increasing attention has been paid to the mechanism by which fungi respond to heat stress. Signal transduction pathways are the key components of heat stress responses in fungi. Therefore, the study of fungi responding to heat stress can provide a basis for the mechanism of the heat stress response in fungi.

Nitric oxide (NO) is a simple small gaseous molecule that can easily diffuse through the cell membrane and that plays an important role in various organisms (5, 6). In plants, NO orchestrates a wide range of processes. NO plays a signaling role in disease resistance (7) and a protective role in the oxidative stress induced in plants by abiotic stresses such as UV-B radiation (8), salt stress (9), and high temperature (10). The accumulated evidence supports the fact that NO can counteract the oxidative environment that forms as a result of ROS production during stress by improving the antioxidant capacity of plants, thus contributing to general plant cell redox homeostasis (11). Therefore, NO is also considered a broad-spectrum antistress molecule (12). NO has also been widely studied in fungi (13). Previous studies have shown that NO can be produced in fungi (14) and may act as a signal molecule when pathogenic fungi infect plants. In addition, the role of NO in fungal morphogenesis and reproduction has also attracted interest and been studied (15). In recent years, NO has been reported to reduce oxidative damage in *P. ostreatus* mycelia by regulating trehalose accumulation (16). Recent studies have shown that NO can regulate ganoderic acid biosynthesis by the calcium-calmodulin interaction in Ganoderma lucidum under heat stress (17). However, in contrast to plants, for edible fungi, the understanding of the biological role of NO is limited. Thus, in-depth investigations to elucidate the mechanism of NO-mediated signal transduction in fungal systems are warranted.

Previous studies have reported that aconitase (ACO) is a major NO target (18). The inhibition of ACO in mitochondria can affect energy metabolism (19). Since mitochondria are the main location of ROS production and accumulation under various stresses, it is speculated that inhibition of ACO in mitochondria would reduce energy metabolism and ROS production. NO can participate in the process of metal nitrosylation and affect the posttranslational modifications of molecules. Specifically, NO can bind to most transition metals in the form of ions, such as iron (Fe^2+^ or Fe^3+^), copper (Cu^2+^), or zinc (Zn^2+^), to form metal-nitrosyl complexes (20). ACO contains a Fe-S cluster that participates in the mechanism of the interconversion of the Krebs tricarboxylic acid (TCA) cycle, and its main function is to catalyze the reversible isomerization of citrate to isocitrate via the intermediate product cis-aconitate. It is suggested that NO may affect the production of energy metabolism by regulating ACO.

Alternative oxidase (AOX) directly couples the oxidation of ubiquinol with the reduction of O_2_ to H_2_O. AOX has emerged as an important mitochondrial component of plant stress responses (21). Previous studies have proved that AOX plays an important role in temperature stress (22), salt stress (23), and drought stress (24) and that AOX also plays a role in maintaining mitochondrial function under environmental adversities (25). In addition, some studies have shown that AOX plays a role in maintaining oxygen homeostasis within mitochondria and also helps reduce the free oxygen concentration, thereby decreasing the production of ROS inside mitochondria (26, 27).

To investigate the protective effect and regulatory pathway of NO in the response of *P. ostreatus* to heat stress, the effects of NO on the gene and protein expression of ACO were confirmed by the exogenous addition of NO donors, such as sodium nitroprusside (SNP) and the NO scavenger cPTIO [2-(4-carboxyphenyl)-4, 4, 5, 5-tetramethylimidazoline-1-oxyl-3-oxide]. In addition, the effects of *aco* mutants on citric acid (CA) content under heat stress were also proven. Subsequently, the effect of CA on the expression of the *aox* gene was explored by the addition of CA, and the function of *aox* in the heat stress response was further verified by the construction and use of overexpression strains.

## RESULTS

### NO production induced by high temperature in *P. ostreatus*.

**FIG 1:**
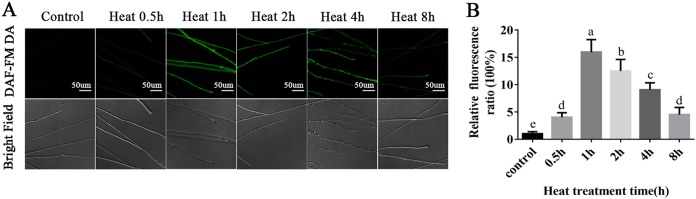
The NO levels were increased in the WT under heat stress. (A) Change in NO level detected using DAF-FM DA staining in the WT with different heat stress times. (B) Changes in the NO fluorescence ratio in the WT strain. The values are the means ± SE of three independent experiments. Different letters indicate significant differences for the comparison of samples (*P* < 0.05 according to Duncan’s test).

The NO content in mycelia changed regularly after heat stress. Fig. 1A shows that the fluorescence intensity of NO first increased and then decreased with the prolongation of heat stress time. Fig. 1B shows that the content of NO in the mycelium was significantly higher after heat stress than under the control conditions, which indicates that heat stress can induce NO accumulation.

### Exogenous NO enhances the resistance of mycelia to heat stress.

**FIG 2:**
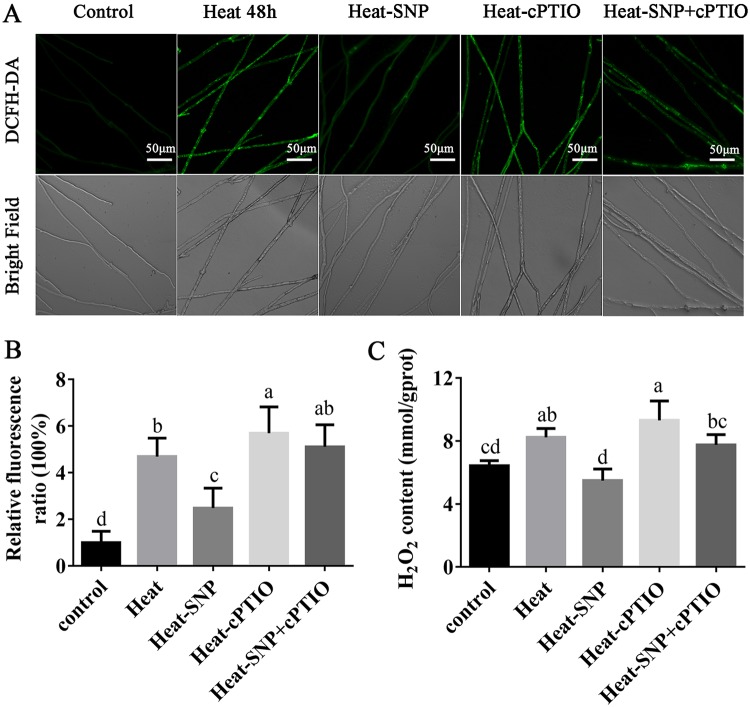
The content of ROS and H_2_O_2_ in mycelia can be regulated by the addition of exogenous NO. (A) Change in ROS levels with 100 μM SNP and 250 μM cPTIO treatment, as detected by DCFH-DA staining. (B) Changes in the ROS fluorescence ratio with 100 μM SNP and 250 μM cPTIO. (C) H_2_O_2_ content with 100 μM SNP and 250 μM cPTIO. H_2_O_2_ was calculated according to the protein concentration, and gprot is protein amount (in g). The values are the mean ± SE of three independent experiments. Different letters indicate significant differences for the comparison of samples (*P* < 0.05 according to Duncan’s test).

In our previous study, we found that high temperature can activate the accumulation of ROS in *P. ostreatus* mycelia (3) and then cause cell damage, and NO alleviates heat stress-induced oxidative damage in Pleurotus eryngii var. *tuoliensis* (16). Thus, the ROS production of *P. ostreatus* mycelia after treatment with 100 μM SNP and 250 μM cPTIO was analyzed by staining with a 2′,7′-dichlorodihydrofluorescein diacetate (DCFH-DA) probe according our previous study. The fluorescence analysis showed that heat stress induced the production of ROS in the mycelia, and the level of ROS was approximately 5.7-fold higher in the treated groups than in the control groups. The ROS accumulation induced by heat stress was reduced by 53% after treatment with 100 μM SNP. The exogenous NO scavenger (cPTIO) promoted the accumulation of ROS and significantly increased the content of ROS. The SNP-mediated decrease in ROS accumulation was abolished by cPTIO (Fig. 2A and B).

H_2_O_2_, as a main kind of ROS, was also quantitatively detected in the NO-treated samples. As shown in Fig. 2C, the H_2_O_2_ content increased significantly after heat stress, whereas exogenous SNP treatment almost completely attenuated this effect. cPTIO blocked the effect of SNP on H_2_O_2_ accumulation. The results showed that the changes in the H_2_O_2_ content and the fluorescent observations showed a similar trend (Fig. 2C). Together, these results support that NO could regulate the accumulation of ROS in mycelia after heat stress.

**FIG 3:**
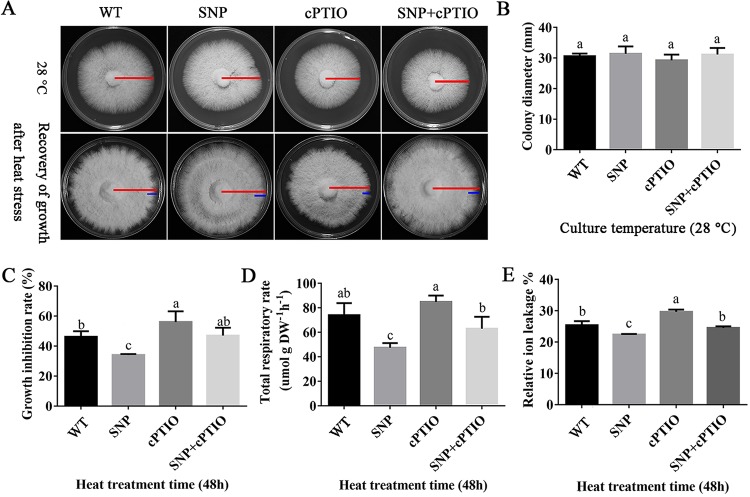
Exogenous NO enhances mycelial resistance to heat stress. (A) The addition of exogenous NO promotes the growth recovery of mycelia under heat stress. Red lines represent the radius of mycelial growth for the entire growth time, and blue lines represent the radius of mycelial growth recovery after heat stress for 48 h. (B) Effect of exogenous NO on colony diameter at 28°C. (C) Effects of exogenous NO on the growth inhibition rate of mycelia under heat stress. (D) Effects of exogenous NO on the total respiration rate of mycelia under heat stress. DW, dry weight. (E) Relative ion leakage after treatment with 100 μM SNP and 250 μM cPTIO. The values are the mean ± SE of three independent experiments. Different letters indicate significant differences for the comparison of samples (*P* < 0.05 according to Duncan’s test).

To study whether NO has a positive effect on the heat stress resistance of *P. ostreatus*, the mycelial colony diameter, growth inhibition rate, relative ion leakage, and total respiratory rate of *P. ostreatus* were measured using potato dextrose agar (PDA) medium containing 100 μM SNP and 250 μM cPTIO. The results showed that after 5 days of culture at 28°C, the addition of 100 μM SNP or 250 μM cPTIO had no significant effect on mycelial growth (Fig. 3A and B). To further explore the protective effect of exogenous NO on mycelia under heat stress, the mycelia cultured for 5 days at 28°C were transferred from PDA plates containing SNP, cPTIO, or both to heat stress treatment. The plates were subjected to heat stress at 40°C for 48 h and then restored to the growing conditions at 28°C for 4 days. The results showed that exogenous SNP promoted the recovery of mycelial growth after heat stress and reduced the inhibition rate of mycelial growth, while exogenous cPTIO showed the opposite effect (Fig. 3A and C).

NO and its derivatives inhibit mitochondrial respiration through various means. To investigate the effect of NO on the respiration of *P. ostreatus*, the total respiration rate of mycelia was measured under heat stress when NO donor or scavenger was added. The results showed that the total mycelial respiration rate decreased significantly after SNP was added and that cPTIO has no significant effect by itself but restores the reduction in the respiration rate caused by SNP. These results suggested that exogenous NO could regulate the respiration rate of *P. ostreatus* mycelium (Fig. 3D). The relative ion leakage rate is one of the indicators of cell membrane damage. Fig. 3E shows the effect of exogenous NO on ion permeability at high temperature. The results showed that after the heat stress treatment, the relative ion leakage rate of the SNP group was 3% lower than that of the wild-type (WT) group, while the relative ion leakage rate of the experimental group with cPTIO was 4.27% higher than that of the WT group. Together, these results suggest that NO attenuates the damage to the mycelial cell membrane under heat stress and that the NO regulation pathway may be related to respiration.

### Exogenous NO inhibits the gene and protein expression of ACO induced by heat stress.

**FIG 4:**
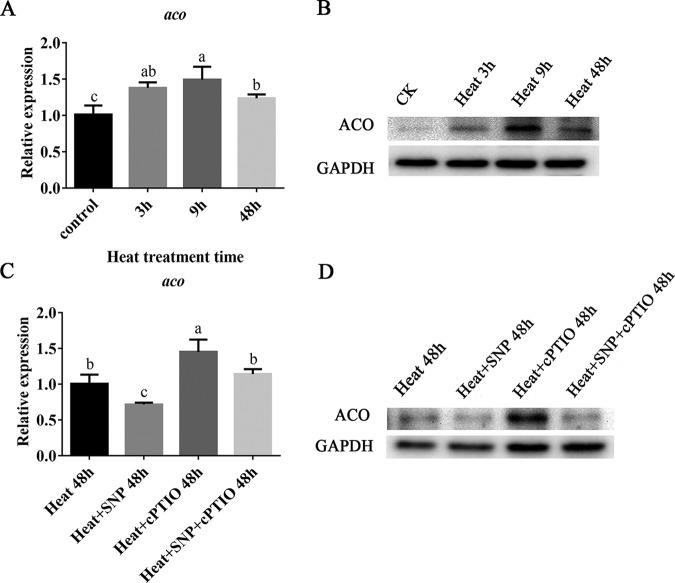
Exogenous NO inhibits the gene and protein expression of ACO induced by heat stress. (A) *aco* gene expression after different lengths of heat stress. (B) Protein expression level of ACO after different lengths of heat stress. (C) Effects of exogenous NO on *aco* gene expression after heat stress. (D) Effects of exogenous NO on the expression of the ACO protein after heat stress. The values are the mean ± SE of three independent experiments. Different letters indicate significant differences for the comparison of samples (*P* < 0.05 according to Duncan’s test).

Fig. 4A shows that the expression of the *aco* gene first increased and then decreased with prolonged heat stress (40°C). After 3 h, 9 h, and 48 h of heat stress, the *aco* gene increased 1.38-fold, 1.49-fold, and 1.24-fold, respectively. As shown in Fig. 4B, the change in the ACO protein level after heat stress was consistent with that at the gene level. SNP, a NO donor, significantly alleviated the increases in the *aco* gene and ACO protein in mycelia induced by heat stress (Fig. 4C and D). To further clarify the role of NO, cPTIO was used, which had a significant effect on both the *aco* gene and ACO protein expression. As shown in Fig. 4C, cPTIO completely blocked the effect of the NO donors on the expression of *aco*, and the relative expression increased more with cPTIO than under heat treatment alone. In Fig. 4D, the addition of cPTIO also significantly promoted an increase in ACO protein, which indicated that cPTIO eliminates the effect of exogenous NO. These results suggest that NO can regulate the expression of the *aco* gene and ACO protein under heat stress. In other words, *aco* is one of the targets of NO in *P. ostreatus*.

### Generation of the OE-*aco* and RNAi-*aco* strains.

**FIG 5:**
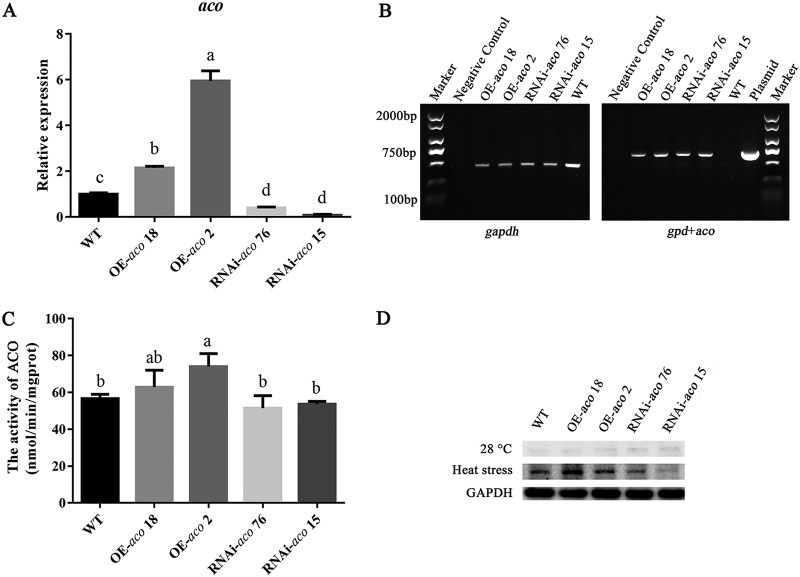
Verification of OE-*aco* and RNAi-*aco* strains. (A) qPCR analysis of the expression of *aco* in the tested strains. (B) PCR assay of the *gpd* and *aco* genes and *gapdh* in *P. ostreatus* transformants, WT, and plasmid. (C) Determination of ACO activity in the tested strains. The activity of ACO was calculated according to the protein concentration, and mgprot is protein amount (in mg). (D) Protein expression level of ACO in the tested strains. Three independent biological replicates were performed for all experiments. The values are the mean ± SE. Different letters indicate significant differences for the comparison of samples (*P* < 0.05 according to Duncan’s test).

To analyze the effect of the *aco* gene on the heat stress resistance of *P. ostreatus*, *aco* overexpression (OE-*aco*) and RNA interference (RNAi)-*aco* vectors were constructed (see Fig. S1 in the supplemental material) and transformed into *P. ostreatus* CCMSSC 00389 mycelium by *Agrobacterium*-mediated transformation. To confirm the identity of transformants, we first amplified the fusion fragment of the *gpd* and *aco* genes (*gpd+aco*) using PCR. Second, the expression of the *aco* gene was further determined by quantitative PCR (qPCR), and the transformants were obtained. As shown in Fig. 5B, a fragment with a length of 775 bp was observed in all transformants analyzed. Fig. 5A shows the detection of the *aco* gene expression in transformants and WT strains by qPCR. The results showed that the expression of the *aco* gene in the overexpression strains OE-*aco* 18 and OE-*aco* 2 was 2.14-fold and 5.95-fold higher, respectively, than that in the WT strain. In the RNAi-*aco* 76 and RNAi-*aco* 15 strains, the expression of the *aco* gene was downregulated by 60.3% and 91.8%, respectively, compared with the WT strain. Fig. 5C was used to determine the ACO enzyme activity of the transformed strains and the WT strain. The results showed that the activity of the ACO enzyme was increased in the overexpression strains compared with the WT strain, while the activity of interference strains was not significantly decreased compared with that of the WT strain. Fig. 5D was used to determine the protein expression of ACO in transformed and WT strains by Western blotting. The results showed that the protein expression of ACO in the overexpression strains was higher than that in WT strain, whereas the effect on interference strains was the opposite. These results suggest that the overexpression and interference of *aco* not only affect gene expression, but also affect the expression of ACO protein.

### *aco* participates in responses of mycelia to heat stress.

**FIG 6:**
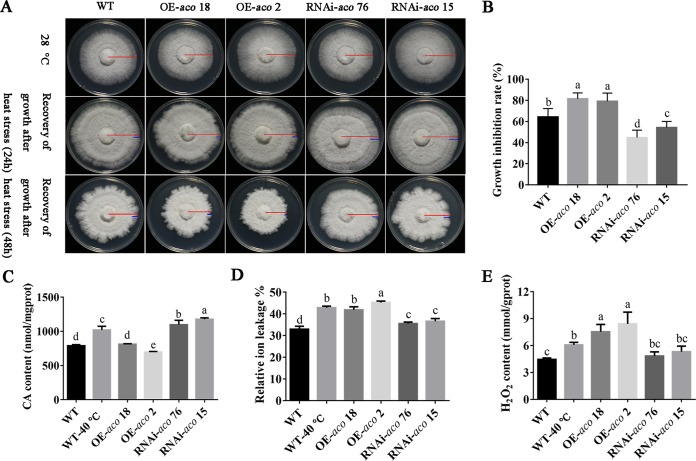
*aco* participated in the response of *P. ostreatus* mycelia to heat stress. (A) Growth recovery conditions of the WT, OE-*aco*, and RNAi-*aco* strains after heat stress. Red lines represent the radius of mycelial growth over the entire growth time; blue lines represent the radius of mycelial growth recovery after heat stress. (B) Mycelial growth inhibition rates of the tested strains after heat stress. (C) CA content in tested strains. (D) Relative ion leakage of the tested strains after heat stress. (E) H_2_O_2_ content of the tested strains after heat stress. The CA and H_2_O_2_ contents were calculated according to the protein concentration, and mgprot and gprot represent protein amounts (in mg and g, respectively). Three independent biological replicates were performed for all experiments. The values are the mean ± SE. Different letters indicate significant differences for the comparison of samples (*P* < 0.05, according to Duncan’s test).

The effect of increased or reduced *aco* gene expression on mycelial growth under heat stress conditions was investigated. Fig. 6A shows that the mycelial growth rate of *aco*-transformed strains was not affected at 28°C compared with that of the WT strain. The mycelial growth inhibition rates of the transformants OE-*aco* 18 and OE-*aco* 2 under heat stress were 81.39% and 79.16%, respectively, which were considerably higher than that observed in the WT strain (64.30%). The mycelial growth inhibition rates of the transformants RNAi-*aco* 76 and RNAi-*aco* 15 were decreased to 44.79% and 54.29%, respectively (Fig. 6A and B). To validate the role of *aco* under heat stress, we detected the change in CA content in transformants. The results showed that the content of CA in OE-*aco* strains was significantly lower than that in the WT strain under heat stress. However, in the RNAi-*aco* strains, the content of CA was increased (Fig. 6C). This finding indicated that *aco* could regulate the content of CA. Fig. 6D shows the change in the relative ion leakage from mycelia after heat stress in the tested strains. The results showed that the relative ion leakage of mycelia increased significantly after heat stress in the WT strain. There was no significant difference in relative ion leakage between the overexpression strains and the WT strain after heat stress. The relative ion leakages of the transformants RNAi-*aco* 76 and RNAi-*aco* 15 under heat stress were 35.51% and 36.60%, respectively, which were considerably lower than that observed in the WT strain (42.87%). Furthermore, the mycelial H_2_O_2_ content of the transformants OE-*aco* 18 and OE-*aco* 2 was significantly increased compared to that of the WT strain under heat stress conditions, while the H_2_O_2_ content of the transformants RNAi-*aco* 76 and RNAi-*aco* 15 was greatly decreased (Fig. 6E). Together, these results suggest that *aco* plays a negative role in heat stress.

### Exogenous CA enhanced mycelial resistance to heat stress.

**FIG 7:**
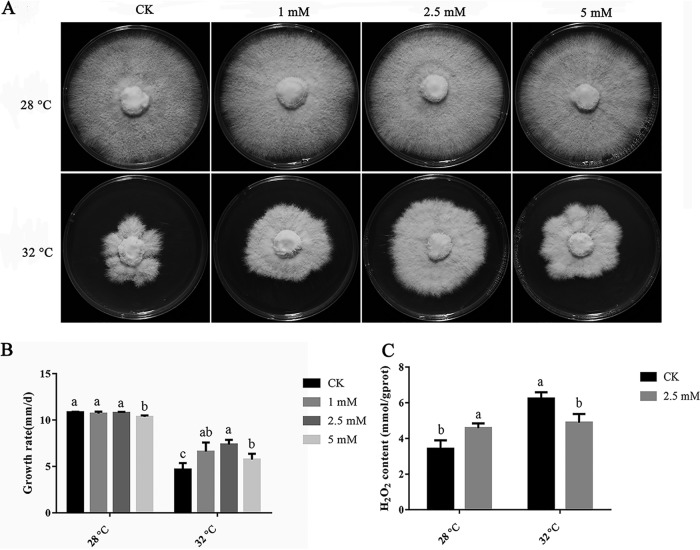
CA-enhanced mycelial resistance to heat stress. (A) Effects of exogenous CA on mycelial growth at 28°C and 32°C. (B) Effects of exogenous CA on the mycelial growth rate of the WT strains at 28°C and 32°C. d, day. (C) Effects of exogenous CA on the H_2_O_2_ content of the WT strain at 28°C and 32°C. H_2_O_2_ was calculated according to the protein concentration, and gprot is protein amount (in g). Three independent biological replicates were performed for all experiments. The values are the mean ± SE. Different letters indicate significant differences for the comparison of the effects of different CA concentrations at 28°C and 32°C (*P* < 0.05 according to Duncan’s test).

To study whether CA has a positive effect on the heat stress resistance of *P. ostreatus*, the mycelial colony diameter and growth rate were measured using media containing different concentrations of CA (0 mM, 1 mM, 2.5 mM, and 5 mM). The results showed that 1 mM and 2.5 mM CA had no significant effect on the mycelial colony diameter and growth rate after 7 days of incubation at 28°C. The addition of 5 mM CA slowed the mycelial growth rate (Fig. 7A and B). Compared with the control check (CK) (0 mM), increased exogenous CA increased the colony diameter and mycelial growth rate at 32°C. When 2.5 mM CA was exogenously added, the mycelial growth rate increased significantly (Fig. 7A and B). In conclusion, exogenous CA enhanced mycelial resistance to heat stress. To further explore the protective effect of exogenous CA on mycelia under heat stress, the content of H_2_O_2_ was detected when exogenous CA was added at a concentration of 2.5 mM. Fig. 7C shows that the content of H_2_O_2_ in mycelia increased significantly at 32°C, and this effect was restored by exogenous CA treatment. Therefore, CA can affect the content of H_2_O_2_ under heat stress.

### CA can induce *aox* gene expression.

**FIG 8:**
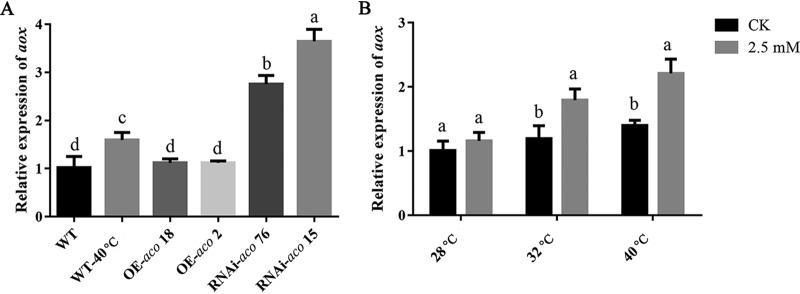
CA-induced *aox* gene expression under heat stress. (A) Effects of *aco*-transformed strains on *aox* gene expression under heat stress. Different letters indicate significant differences between strains. (B) Effects of exogenous CA on the relative expression of *aox* at 32°C and 40°C. Three independent biological replicates were performed for all experiments. The values are the mean ± SE. Different letters indicate significant differences for the comparison of samples with 2.5 mM CA at 28°C, 32°C, and 40°C (*P* < 0.05 according to Duncan’s test).

To further explore the pathway that responds to CA under heat stress, the expression of the *aox* gene was detected. Fig. 8A shows that the overexpression of *aco* downregulated the expression of the *aox* gene under heat stress, which was contrary to the effect on RNAi-*aco* strains. This trend is consistent with the effect of CA on *aco*-transformed strains. Fig. 8B shows that the addition of exogenous CA caused the expression of the *aox* gene to change slightly at 28°C, but the addition of exogenous CA significantly increased the expression of the *aox* gene under heat stress (Fig. 8B). Taken together, these results show that CA can activate the expression of the *aox* gene under heat stress.

### Overexpression of the *aox* gene increases mycelial resistance to heat stress and H_2_O_2_.

**FIG 9:**
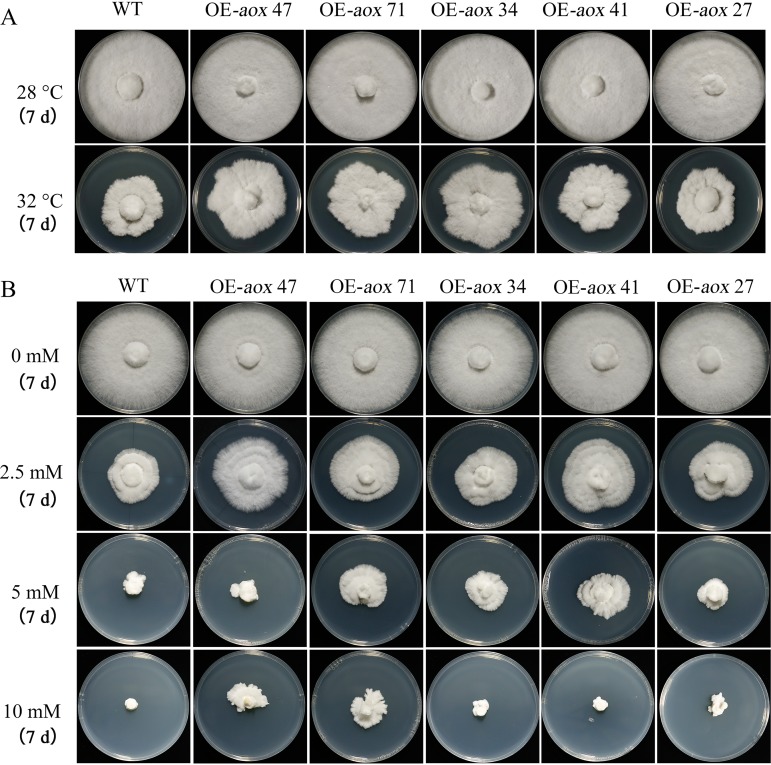
OE-*aox* strains enhanced the resistance of mycelia to heat stress and exogenous H_2_O_2_. (A) Colony morphology at different temperatures (28°C and 32°C). (B) The effects of exogenous H_2_O_2_ at different concentrations on mycelial growth. d, days.

In order to explore whether the upregulation of *aox* gene expression enhances the resistance of mycelia to heat stress, OE-*aox* strains were constructed, and the validation results are shown in Fig. S2. The *aox* gene sequence is shown in the supplemental material. Fig. 9A shows the growth status and rate of the tested strains. The results showed that the edges of colonies were irregular when the mycelium growth temperature was 32°C, while the colony diameter of OE-*aox* strains showed obvious advantages. The colony diameters of OE-*aox* 47, OE-*aox* 71, and OE-*aox* 34 were significantly larger than that of the WT.

Heat stress can lead to a significant increase in H_2_O_2_ in mycelia. To investigate whether the *aox* gene is involved in the ROS response, the growth rates of the WT and OE-*aox* strains were evaluated on PDA plates with different concentrations of exogenously added H_2_O_2_. The results suggest that with the increase of exogenous H_2_O_2_ concentration, the inhibition of mycelium was also enhanced. When the concentration of exogenous H_2_O_2_ was 2.5 mM, the colony diameter of OE-*aox* strains was increased compared with that of the WT strain. When the concentration of H_2_O_2_ was increased to 5 mM, the colony diameters of OE-*aox* 71, OE-*aox* 41, and OE-*aox* 34 were significantly higher than that of the WT strain. When the concentration of H_2_O_2_ was 10 mM, the WT strain stopped growing, while the OE-*aox* 47 and OE-*aox* 71 strains germinated (Fig. 9B). In conclusion, the overexpression of the *aox* gene enhanced mycelial tolerance to H_2_O_2_.

## DISCUSSION

Previous studies found that NO pretreatment reduced the damage from heat stress to rice seedlings (28) and increased the survival rate of wheat leaves and maize seedlings (29). NO can activate antioxidant enzymes and reduce ROS levels under heat stress (30). The role of NO in fungi has also been extensively studied. In Candida albicans, NO is involved in oxidative stress and azoles (31). In Trichoderma harzianum, NO can alleviate oxidative damage (32), and this effect was also demonstrated in P. eryngii var. *tuoliensis* (16). In this study, with the prolongation of heat stress treatment time, NO content in mycelia increased regularly. The results showed that heat stress induced an increase in NO content in mycelia. Under heat stress, the addition of exogenous SNP alleviated the excessive production of ROS in mycelia, reduced relative ion leakage, and promoted the recovery of mycelium growth after heat stress. The protective effect of SNP on mycelia was blocked by the addition of cPTIO. Previous studies have shown that NO alleviated heat stress through the regulation of trehalose accumulation and ROS-scavenging enzymes in P. eryngii var. *tuoliensis* (16), and transcriptome data showed that NO improved the resistance of Ganoderma oregonense to heat stress (33). NO may reduce ROS content by regulating unknown pathways that decrease cell membrane damage, thereby reducing extracellular ion concentrations and promoting mycelial growth. Our results confirmed that NO can enhance the resistance of *P. ostreatus* to heat stress by regulating ACO.

ACO, a TCA cycle enzyme localized in mitochondria, is a prototypical example of a multifunctional protein (34) that can catalyze the reversible isomerization of citrate to isocitrate (35). Previous studies have shown that one of the primary targets of NO in mitochondria is ACO. ACO is an iron-sulfur (4Fe-4S) cluster-containing enzyme, which makes it very sensitive to ROS and active nitrogen. In tobacco, it was shown that NO produced by tobacco mosaic virus attack inhibited ACO (36). In this study, we demonstrated that ACO protein in mycelia increased first and then decreased with the prolongation of heat stress treatment time using Western blotting. It is speculated that ACO may be involved in the response of mycelia to heat stress. The expression of the *aco* gene and ACO protein was significantly downregulated after SNP was added. In contrast, the addition of exogenous cPTIO significantly increased the expression of the ACO protein and the *aco* gene. This result suggested that NO produced under heat stress inhibited ACO protein and *aco* gene expression in *P. ostreatus*. This is consistent with previous research results. These results suggest that NO regulates the expression of the *aco* gene and ACO protein in *P. ostreatus*. A “circuit breaker” mechanism has been proposed in which ACO inactivation leads to reduced electron flow through the mitochondrial electron transport chain and to a subsequent decrease of ROS (37, 38). It is presumed that this regulatory mechanism exists in *P. ostreatus*.

In order to explore the regulation pathway that alleviates the oxidative damage in mycelia when ACO is inhibited by NO, OE-*aco* and RNAi-*aco* strains were constructed. In this study, RNAi-*aco* strains showed significant advantages in restoring growth after heat stress. Moreover, the content of H_2_O_2_ and ion permeability were decreased after *aco* interference. The results indicated that when *aco* was inhibited, it could enhance the resistance of mycelia to heat stress. This suggests that *aco* plays a negative role in the response to heat stress in *P. ostreatus*. The substrate of the ACO enzyme is CA. CA, one of the intermediate products of TCA, is a source of carbon skeleton and cell energy, which are used in the respiratory cycle and other biochemical pathways. CA is a vital organic acid that has been reported to be closely related to aluminum poisoning (39), iron stress (40), and salinity stress (41). Furthermore, in this study, the results showed that *aco*-transformed strains could significantly affect CA content under heat stress. Therefore, *aco* can further regulate the heat stress tolerance of *P. ostreatus* mycelia by regulating the endogenous CA content in mycelia. Previous studies have shown that under aluminum stress, the inhibition of ACO resulted in an increase in CA content in maize roots (42). Our results are similar to those of previous studies. In addition, the addition of exogenous CA further proved that CA could enhance the tolerance of mycelia to heat stress and reduce the content of H_2_O_2_ in mycelia. In Lolium arundinaceum, exogenous CA application may alleviate the growth inhibition and physiological damage caused by high temperature (43). This finding indicated that CA was involved in the heat stress response.

Alternative respiratory pathways include a single protein, AOX, which plays important roles when the cytochrome pathway is inhibited under various stress conditions (44). Previous studies have shown that AOX protein does not contribute to proton gradient formation but reduces the uncontrolled leakage of electrons to oxygen when the ubiquinone pool is overreduced under stress (45). Thus, the induction of AOX results in avoidance of ROS generation via prevention of overreduction of the ubiquinone pool (46). In this study, we found that endogenous CA (RNAi-*aco* strains) and exogenous CA induced *aox* gene expression under heat stress. A large amount of evidence has suggested that the enhanced alternative pathway improved stress tolerance by maintaining redox homeostasis in the plants (21, 47). In fungi, AOX is involved in stress defense mechanisms or in metabolism regulation. For example, in Sclerotinia sclerotiorum, AOX participates in the regulation of growth, development, and responses to oxidative stress (48). In Aspergillus fumigatus and Neurospora crassa, cyanide-insensitive respiration catalyzed by AOX is induced by heat shock and oxidative stress (49, 50). This study showed that the resistance of OE-*aox* strains to heat stress was significantly stronger than that of the WT strain. In addition, overexpression of *aox* significantly enhanced mycelial tolerance to H_2_O_2_. This suggests that CA could enhance the high temperature tolerance of *P. ostreatus* mycelia by inducing *aox*. This result is similar to those of previous studies. In addition, previous studies have proven that increased AOX activity can reduce the production of O_2_^−^. This further reduces the conversion of O_2_^−^ to other ROS (51). Based on the results of this study, it is speculated that the increase in CA content in *P. ostreatus* can induce *aox* gene expression under heat stress. The high expression of *aox* changes the respiratory pathway under heat stress, reduces the production of ROS in the respiratory chain, and further reduces the oxidative damage of mycelia.

**FIG 10:**
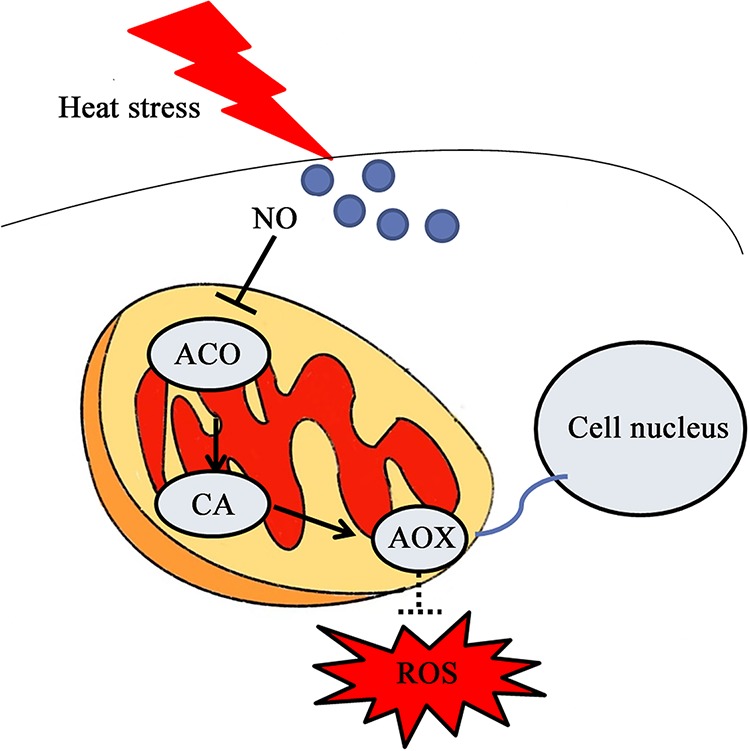
Schematic representation of how NO regulates the response of *P. ostreatus* to heat stress by inhibiting *aco*.

In conclusion, our data suggest that NO inhibits the expression of the ACO protein and *aco* gene under heat stress. Construction and characterization OE-*aco* and RNAi-*aco* strains showed that the inhibition of ACO increases the accumulation of CA in mycelia. Further experiments showed that an increase in CA induces *aox* gene expression. Finally, *aox* overexpression enhanced the resistance of mycelia to heat stress and H_2_O_2_. Based on these findings, a potential cascade of cellular events constituting the NO-mediated signaling pathway was proposed (Fig. 10). Our study improves the understanding of the biological functions and regulatory pathways of NO in *P. ostreatus* under heat stress. In the future, we will further explore how NO regulates ACO and the specific regulatory role of the *aox* gene in stress.

## MATERIALS AND METHODS

### Strain and growth conditions.

*P. ostreatus* P89 (CCMSSC 00389) was provided by the China Center for Mushroom Spawn Standards and Control. The WT, OE-*aco*, and RNAi-*aco* strains were cultured on PDA plates for growth tests and recovery growth tests after heat stress. Agrobacterium tumefaciens GV3101 (IMCAS, Beijing, China) was grown in Luria-Bertani (LB) medium (Oxoid, England) containing 100 μg/ml kanamycin (VWR Life Science, USA) and 50 μg/ml rifampin (MP Biomedicals, France) and was used to transform *P. ostreatus*. Escherichia coli DH5α and BL21(DE3) (Tiangen, Beijing, China) were used for plasmid construction and were grown in LB broth containing kanamycin (50 μg/ml).

### NO and ROS detection assay.

According to a previous method, intracellular NO production was measured using the fluorescence probe 4-amino-5-methylamino-2′,7′-difluorofluorescein diacetate (DAF-FM-DA). Mycelia were stained with DAF-FM-DA for 30 min, and NO was detected with a laser scanning confocal microscope (Carl Zeiss LSM700, Oberkochen, Germany) (14). The ROS concentration was assessed according to a previously described method, and intracellular ROS production was measured by the fluorescence probe DCFH-DA (52). The fluorescence was analyzed using Zeiss software (ZEN lite, Zeiss, Göttingen, Germany) (53).

### Construction of OE and RNAi plasmids and strains.

Gene transformation with a gene knockout and overexpression vector is a useful approach to explore the function of genes in fungi (54). The construction of a fungal RNAi vector and overexpression (OE) vector was performed as previously described. Target gene fragments were amplified by the primers shown in Table 1. Homologous recombination was then used to connect the target fragment to the plasmid. The constructed plasmid map is shown in Fig. S1. Then OE and RNAi vectors were transformed into *P. ostreatus* using A. tumefaciens GV3101 according to our previous study. The *aox* and *aco* gene sequences are provided in the supplemental material.

**TABLE 1:** Primers used in this study

Primer^*a*^	Sequence (5′→ 3′)	Note
Po-*gpd*-F	GGTACCTTTATTGGCGGT	Promoter cloning
Po-*gpd*-R	CCAGGTCAGTGAAATTTCC	
*aco*_g-F	ATGTTCAACTGCGACCG	gDNA fragment cloning
*aco*_g-R	CTAGTTGCCCTTGGCCTTAGC	
*aco*_c-F	ATGTTCAACTGCGACCG	cDNA fragment cloning
*aco*_c-R	CTAGTTGCCCTTGGCCTTAGC	
*aox*_g-F	ATGCTTCGCGCGCAACTC	gDNA fragment cloning
*aox*_g-R	TCAATTACTACCTTTCTGCTTCTCT	
*aox*_c-F	ATGCTTCGCGCGCAACTC	cDNA fragment cloning
*aox*_c-R	TCAATTACTACCTTTCTGCTTCTCT	
*aco*-q-F	CCTCACCGTTCTCAATGTT	qPCR
*aco*-q-R	CGACGAAGGCATGAGTAG	
*aox*-q-F	CTCCACAGATCCACACTCTTC	
*aox*-q-R	CCGCCTCAAAGTCATAAAAGTC	
*β-actin*-F	GCGATGAACAATAGCAGGG	Endogenous control
*β-actin*-R	GCTGGTATCCACGAGACAAC	
*aco*-OE-F	TTACAGGTCAAAGTTATGTTCAACCTAGGAACCG	Construction of OE plasmids
*aco*-OE-R	AATTCTAGAGG GCCCCTAGTTGCCCTTGGCCTTAGC	
*aox*-OE-F	GGTCAAAGTTACTAGTATGCTTCGCGCGCAACTC	
*aox*-OE-R	CAATTCTAGAGGGCCCTCAATTACTACCTTTCTGCTTCTC	
*aco*-RNAi-F1	CTTTACCATCTCCTCAGATCTAACTTGTCAGAACTCGAGCCACA	Construction of RNAi plasmids
*aco*-RNAi-R1	GGAGCTAAGCTCTAAACTAGTCTGCGAGTGACTCGGTGAGC	
*aco*-RNAi-F2	TTCTTCTATTATAAGACTAGTTAGAGTTGGGTTCGCCCTTCT	
*aco*-RNAi-R2	CATGCCAATTCTAGAGGGCCCAACTTGTCAGAACTCGAGCCACA	
*gpd+aco*-F	CTACTACTCCTTGACCGCTGAT	Detection of transformants
*gpd*+*aco*-R	AATCCTTGACACCACCAACTTG	
*hyg*-F	CGACAGATCCGGTCGGCATCTACTCTATTTCTT	
*hyg*-R	TCTCGTGCTTTCAGCTTCGATGTAGGAGGG	
*gapdh*-F	ATCCATATCTTCGCTGAGAA	
*gapdh*-R	GGACGGAATAATGTTGTTGT	

a F, forward; R, reverse.

### RNA extraction, reverse transcription, and qPCR.

The levels of specific mRNAs expressed by the WT and transformed strains were assessed using qPCR according to methods in a previous study (3). A fusion fragment containing the *gpd* promoter and *aco* gene was amplified using the primer pair *gpd*+*aco*-F and *gpd*+*aco*-R (Table 1). Based on a previous description, total RNA was extracted using the E.Z.N.A. plant RNA kit (Omega Bio-tek, Norcross, GA, USA) following an extraction method for fungal samples. First-strand cDNA was synthesized using the HiScript II 1st strand cDNA synthesis kit (Vazyme, Nanjing, China) according to the manufacturer’s instructions. The ChamQ SYBR qPCR master mix kit (Vazyme, Nanjing, China) and the ABI 7500 real-time PCR amplifier (Applied Biosystems, Foster City, CA, USA) were used for qPCR. The qPCR amplification procedure was as follows: 95°C for 3 min, 40 cycles at 95°C for 3 s and at 60°C for 32 s, and a final extension at 72°C for 30 s. Relative gene expression was analyzed according to the 2^−△△CT^ method (55).

### Heat stress treatment.

The WT, OE-*aco* 18, OE-*aco* 2, RNAi-*aco* 76, RNAi-*aco* 15, and OE-*aox* strains were used in this study. High temperature is one of the abiotic stresses that impact the cultivation of *P. ostreatus*. To simulate heat stress in growth, the strains were cultured and treated according to our previous methods (55). The mycelia were cultured at 28°C for 5 days, treated at 40°C, and then returned to growth at 28°C. To further explore whether exogenous CA and *aox* genes can enhance mycelial resistance under heat stress, the tested strains were inoculated and cultured directly on PDA plates at different temperatures (28°C and 32°C) for 7 days.

### Determination of mycelial growth inhibition rate.

Using our previous research methods (3), the mycelial growth inhibition rate was determined. First, the average growth rate (A1) of the strain was measured at 28°C for 5 days. Then, mycelia were heat stressed and placed at 28°C for recovery growth. The mycelial growth rate (A2) was measured during the recovery process. The mycelial growth inhibition rate is (1 – A2/A1) × 100%.

### ACO activity assay and CA content determination.

The WT, OE-*aco*, and RNAi-*aco* strains were cultured on PDA plates for 5 days and under heat stress conditions for 2 days. Subsequently, the mycelia were quickly scraped, mixed, and frozen in liquid nitrogen for further use. Following the manufacturer’s instructions, the activity of ACO was determined by using an ACO activity detection kit, and the content of CA was determined by using a CA content detection kit (Suzhou Keming Biotechnology, Suzhou, China).

### Western blotting.

The expression of ACO protein in the WT and transformed strains was studied using Western blotting with ACO polyclonal antibody as a target. Western blot analysis was performed according to the method of a previous study (55). Briefly, equal amounts of total protein (25 μg) were loaded into the lanes, separated in a 10% (wt/vol) sodium dodecyl sulfate PAGE gel, and transferred to polyvinylidene difluoride membranes. Western blot analysis was performed using antibodies against ACO (GenScript, Nanjing, China), and glyceraldehyde 3-phosphate dehydrogenase (GAPDH; JGI database accession no. PC15_1090663) was used as a control.

### Determination of H_2_O_2_ concentration.

Intracellular H_2_O_2_ content was determined using a hydrogen peroxide assay kit (Nanjing Jiancheng Bioengineering Institute, Nanjing, China) according to the manufacturer’s instructions. H_2_O_2_ reacts with molybdic acid to form complex. The amount of complex formation was determined by measuring the absorbance at 405 nm, and the content of H_2_O_2_ was calculated.

### Determination of relative ion leakage and total respiratory rate.

Ion leakage was detected according to the method of previous studies with modifications (56). Ten pellet pieces (5 mm) were inoculated into 100 ml of potato dextrose broth (PDB) medium and cultured for 5 days at 28°C with shaking at 180 rpm. Heat stress was then applied for different lengths of time at 40°C. The conductivity of mycelium pellets (C1) was measured by washing the electrolytes attached to the surface with deionized water and then putting them into 20 ml of deionized water at 28°C for 2 h. Then, the sample was autoclaved for 30 min to determine the total conductivity (C2). The relative ion leakage rate (%) was C1/C2 × 100%. The total respiratory rate was measured according to previous studies (57).

### Statistical analysis.

GraphPad Prism 6 (GraphPad Software, Inc., San Diego, CA, USA) was used for statistical analysis. The values are reported as the mean ± standard error (SE) and were analyzed using one-way analysis of variance (ANOVA), with a *P* value of <0.05 considered significant.

## Supplementary Material

Supplemental file 1
