# Supplementary material for: Nitric Oxide Improves the Tolerance of Pleurotus ostreatus to Heat Stress by Inhibiting Mitochondrial Aconitase
Source: Appl Environ Microbiol. 2020 Feb 18;86(5):e02303-19. doi: 10.1128/AEM.02303-19 (PMC7028963; doi:10.1128/AEM.02303-19)
Supplement: Supplemental file 1 [file AEM.02303-19-s0001.pdf]

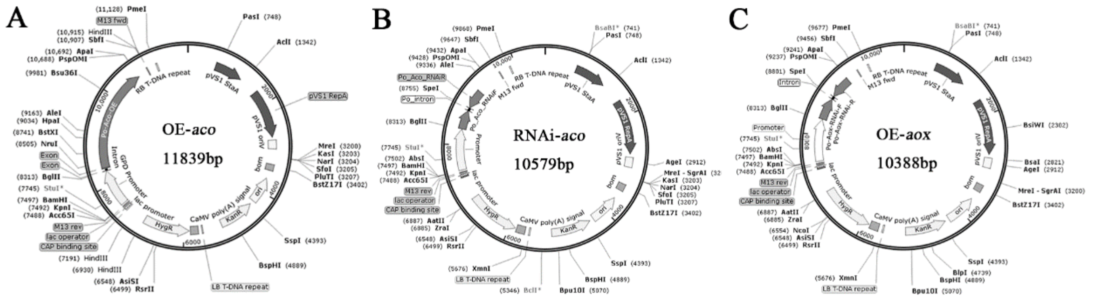

Supplemental Fig. S1 Strategy for the overexpression and RNA interference of target genes in *P. ostreatus*. A. The structure of the OE-*aco* vector. B. The structure of the RNAi-*aco* vector. C. The structure of the OE-*aox* vector.

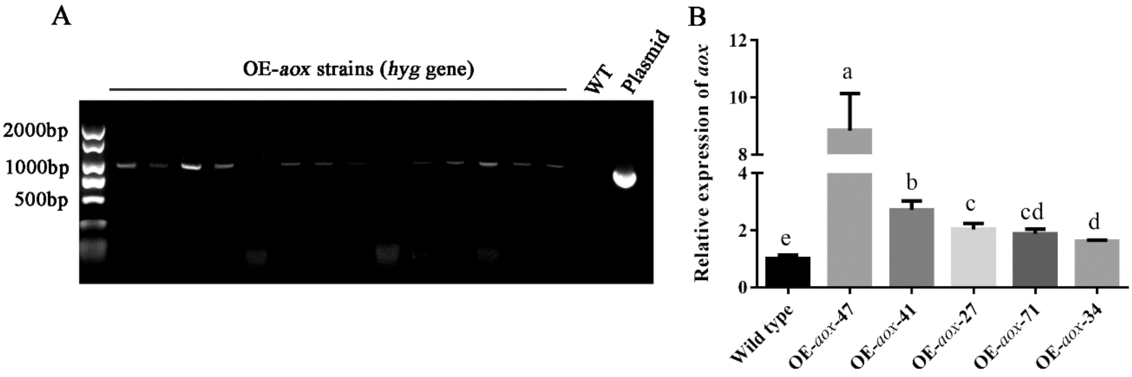

Supplemental Fig. S2 Validation and selection of OE-*aox* strains. A. Preliminary selection of OE-*aox* transformation strains by amplification of the *hyg* gene. B. qPCR analysis of the expression of *aox* in the tested strains. The values are the mean ± SE. Different letters indicate significant differences for the comparison of samples ($P < 0.05$, according to Duncan's test)

Supplemental gene sequence

>*aco*_cDNA_*Pleurotus ostreatus*_CCMSSC00389

ATGTTCAACCTAGCGAACCGCACTGCCGGTGCTCTTCGCTCGCTGCAGCGCATGGCCG
CCGGCATGGCCACAGCTGCACCGGAGCGAATTGGAGATACCAAAGTTCCTATGAGTTT
GCTTGAAAAGGGCGCATACATCAACTACCAGCGTATAGAGGACAACCTGGTCATCGTT
CGCGATAGATTGAAGCGACCATTGACCCTATCGGAGAAAATTCTTTACGGTCATCTTGA
CGATCCTCATGGACAAGATATTGAACGTGGTGTCTCATATCTCAAGCTGCGGCCTGACC
GAGTCGCCTGCCAGGACGCTACGGCGCAGATGGCTATCTTACAATTCATGTCTGCTGGC
ATGGACACTGCTGCCGTTCCGACCACCGTACACTGTGACCACTTGATTGAGGCCCAAG
TTGGTGGTGTCAAGGATTTGGCCCGCGCTATCGATATCAACAGGGAGGTTTACGACTTT
TTGGCTACTGCTACTGCGAAGTACGGTCTTGGTTTCTGGAAACCCGGCTCTGGTATTAT
CCATCAAATCATTTTGGAGAACTACGCATTCCCTGGTGGTCTTATGATTGGCACTGATTC
GCACACGCCCAACGCTGGTGGCCTCGGTATGATTGCATGTGGAGTTGGAGGTGCTGAT
GCAGTCGACGTCATGGCGGGCCTACCATGGGAACTGAAATGCCCGAAGGTCATTGGTG
TTAACCTTACTGGTAAAATTAGCGGCTGGACTTCCCCCAAAGATGTTATTCTCAAGGTT

GCTGGCATATTGACGGTCAAGGGTGGAACTGGCGCTATCGTCGAATACAAGGGTCCTG
GTGTTGAATCACTCTCGTGCACGGGTATGGCGACTATTTGCAACATGGGTGCTGAGATA
GGCGCAACAACGTCGATGTTCCCATTCAACAACCGCATGGTCGACTATCTAAAGGCCA
CCAAGCGCGGCGACATTGCCAACTATGCTTCTCGCTTCGCACACAATCTCCAGGCTGAT
GAGGGTTCCCAATACGATGAACACATCGAAATTAACTTGTCAGAACTCGAGCCACACG
TCAACGGTCCTTTCACTCCCGATCTCGCCACCCCCATTTCCAAGTTCGCCCAGGCTGTT
GAGGAGAACAAGTGGCCCGCTGAGCTCAAGGTCGCCTTGATCGGTTCTTGCACTAACT
CTTCGTACGAGGATATGTCTCGCTCTGCCTCCATCGCCAACGAAGCCAAGGCACACGG
TCTCTCCGTCGCAAGCAAGTACACCATCACTCCCGGTTCCGAGCAAGTCCGCGCTACC
ATCGCCCGTGACGGTCAAATGGCTGCTCTTGAGGATGTTGGAGGCTTGGTTTTGGCCA
ACGCCTGCGGTCCTTGCATCGGTCAATGGGACAGGAAGGATGTTAAGAAGGGCGAAC
CCAACTCTATCATCACTTCTTACAACCGTAACTTCACTGGCCGTAACGATGCTAACCCC
GCTACTCATGCCTTCGTCGCCTCCCCCGATATCGTGACCGCCATGGCCTTCGCTGGCGA
CCTCCGTTTCAATCCCCTCACTGACTCCCTCAAGGGTGCCGACGGCAAGGAATTCAAG
TTTTCCGCCCCCACTGGCAACGAGCTCCCCCCTCGCGGTTATGACCCCGGTGAAGATA
CCTTCCAACCTCCTCCCGAGGATCGTGCCTCTGTCCAAGTTGCCGTCAGCCCCACTAGT
GACCGTCTTCAGCTGTTGAAGCCCTTCCAGCCATGGGACGGCAAGACACCAAGTGATC
TCCCCATCTTGATCAAGGTCAAGGGCAAGTGCACAACCGATCACATCTCCGCCGGTGG
ACCATGGCTCAAATACCGTGGCCATCTCGAGAACATCTCGCAAAACTGTCTGATTGGTG
CCATCAACATTGAGAACGGTGAGGCCAACAAGGTCAAGAACCAACTTACTGGTGAAT
GGGGAGGAGTCCCCCAGACTGCTGCCTCCTACCGCGAGAAGGGCATCAAGTGGGTCG
TCATTGGTGACCACAACTATGGTGAAGGTTCATCTCGTGAGCACGCCGCCCTCGAGCC
CCGCTTCCTCGGCGGTCTCGCCATCATCGTCCGCAGCTTTGCCCGTATCCACGAAACCA
ACTTGAAGAAGCAAGGCATGCTTGCTTTGACCTTCGTTGACCCCGCCGACTACGACAA
GATTCGTCCAGACGACAAGGTCGATATCCTCGGCCTCGAGGACTTCAAGCCCGGCCAG
AACTTGACTCTCAACGTCAAGCACTCTGACGGCGCCTCCGAGCAGATCCCCCTCGCAC
ACTCCTTCAACGAGGGACAGATTGAGTGGTTCAAGGCTGGCTCAGCATTGAACTTGAT
GGCTGCTAAGGCCAAGGGCAACTAG

>*aox*_cDNA_*Pleurotus ostreatus*_CCMSSC00389

ATGCTTCGCGCGCAACTCCACCAACAGGTATGTAGACAGGTCACAGGACGGTCCTTTG
TCCTGGTCTCTCTGGCTACACCCCTCGCGACTAGTCGCCGAACCTTTTCCACCACACCG
AACCCCTTTAACGCCTTGACAAGTGATGCTGCGAAAGCTGCTGCAACTCCACATCCAA
CTGTTGGGAAGGGCCAGGCGCCACAACGAGCGAGCTCCGCAGACGGAGCAATAACGC
AGTCGACGCCGGTCACCACGGTGCCCACTATGATCAGAGGCGATTGGGTCCTCTTTCA
CCCTGTTTATACGCCCGAAGAGTTGAAGGCGGTGCAGATTCTACACAAAGAGACAAAA
TCTATGACAGATAAGATCGCATACGGTCTTGTCAGATTGGCCAGGGCTGGGTTCGATTT
CGTCTCGAGATATAAACACAAACCCCTTCCTCCTAGCCATAACATGACCCTGCAAGAGC
TTCGCAACGAAGGTTACCTCCTGGATCAGAATGCATGGCTTAACCGAATTCTATTCTTG
GAAACGATCGCAGGTGTGCCCGGTATGGTAGGTGCGACCTTGCGGCATCTACAAAGCC
TACGTTTAATGCGGCGTGACAATGGGTGGATCCACACTCTTCTATCGGAATCGGAAAAC
GAGCGGATGCACTTGATGACTTTTATGACTTTGAGGCGGCCTACACCATTCTTCAGGGC
GTTGGTTCTCGGTGCACAGGGTGTTTTCTACAACTTGCTCTTTTTGACGTACATCGTCT
CACCTCGAGCTTGCCATCGCTTCGTCGGATATCTTGAAGAGGAAGCTGTCATCACTTAT
ACTCGCTGTATACAGGACCTGGAAGCAGGCCGAATCCCTGAATGGGAGAATGAGCCTG

CACCCGAGATCGCCATCGATTACTGGCGCTTGCGCCCGGATGCCAAATTACTCGATGTT
CTCTACGCTGTTCGATCCGATGAAACGACACATCGATTCGTCAACCACAGTCTTGCCGA
CCTGAATACAACAACAGACGTCAATCCTTTCGCATTCAGGGAACCGGATATGCATGTTA
AAGGGAACAAGATTGGATTCGAGCGTTCGGAGTCGGAGAAATATGTCCAGGAATCTCA
TGATCTATATCTTCAAGAGAAGCAGAAAGGTAGTAATTGA
